# A paradigm shift in diagnosis and treatment innovation for mucinous cystic neoplasms of the liver

**DOI:** 10.1038/s41598-024-67320-2

**Published:** 2024-07-17

**Authors:** Bin Shi, Peng Yu, Lingzhan Meng, Hu Li, Zizheng Wang, Li Cao, Jin Yan, Yanling Shao, Ying Zhang, Zhenyu Zhu

**Affiliations:** 1 Department of Organ Transplantation, The Third Medical Center of PLA General Hospital, Beijing, China; 2 Department of Hepatology Surgery, The Fifth Medical Center of the PLA General Hospital, Beijing, China; 3 Department of General Surgery, The Fifth Medical Center of the PLA General Hospital, Beijing, China

**Keywords:** Mucinous cystic neoplasms, Invasive carcinoma, Liver, Diagnosis, Treatment, Surgical resection, Surgical oncology, Outcomes research

## Abstract

This study comprehensively explores the clinical characteristics, diagnostic approaches, and treatment methods for liver mucinous cystic neoplasms (MCN). A retrospective analysis was conducted on seven individuals diagnosed with MCN, admitted to the Fifth Medical Center of the PLA General Hospital between October 2016 and May 2023. Preoperative AFP was negative, while CA19-9 was elevated in two cases. Surgical resection was performed for all patients. The patients showed favorable postoperative recovery. Follow-up revealed an excellent overall survival rate, except for one case of invasive carcinoma resulting in tumor recurrence and metastasis 6 months after surgery. MCN poses a diagnostic challenge due to the absence of distinct clinical and radiological features, leading to potential misdiagnosis and inappropriate treatment. Patients with suspected liver cystic diseases should consider the possibility of MCN. Surgical resection has proven to be a practical approach with satisfactory therapeutic outcomes.

## Introduction

Hepatic mucinous cystic neoplasm (MCN) is a rare closed-type liver tumor that mostly presents benign characteristics, grows slowly, usually lacks communication with normal bile ducts, but carries a certain tendency for malignancy, classified as a precancerous lesion. According to the WHO 2019 classification of digestive system tumors, the diagnostic terms “bile duct adenoma” and “biliary cystadenocarcinoma” are no longer used. Instead, the term “hepatic mucinous cystic tumor” is adopted, categorized into three types: mucinous cystic tumor with low-grade dysplasia, mucinous cystic tumor with high-grade dysplasia, and invasive carcinoma associated with mucinous cystic tumor^1^.

Symptoms of MCN can vary based on tumor location and size, including abdominal pain, jaundice, nausea, vomiting, gastrointestinal upset, weight loss, and abdominal mass^2–5^. However, these symptoms are nonspecific and may overlap with other biliary tract disorders, necessitating further testing for diagnosis^6,7^. Imaging techniques such as ultrasound, CT, and MRI are commonly used to diagnose MCN, assess tumor characteristics, and detect distant metastases^8,9^. Despite the lack of distinguishing clinical and radiological features for MCN, leading to potential misdiagnosis and inappropriate treatment, surgical resection has been proven as a feasible and effective approach^10^. Surgery can be performed through open, laparoscopic, or robotic techniques, aiming for complete tumor removal. Depending on the specific circumstances, partial liver tissue excision may be necessary to achieve satisfactory treatment outcomes.

Treatment options for primary liver tumors include surgery, local therapies, drug therapy, liver transplantation, and comprehensive treatment. Surgical resection is most effective for early-stage tumors. For patients ineligible for surgery, options like tumor ablation and chemoembolization can be considered. Advanced primary liver tumors can benefit from oral targeted drugs and immunotherapy^11–13^. However, limited donor availability restricts liver transplantation. Comprehensive treatment combines surgery, local therapies, and drug therapy to control tumor growth and metastasis, extending survival. Early detection, prompt treatment, and regular follow-up are vital for improved outcomes and prognosis. Identification of prognostic biomarkers is essential for guiding treatment decisions^14^.

This study retrospectively analyzed data from seven MCN cases and conducted a comprehensive literature review to deepen our understanding of these diseases and improve diagnostic and therapeutic efficacy in their management.

## Materials and methods

### Clinical data

For inclusion in this study, patients analyzed by the author had to meet three criteria:

1.1. Admitted to the Hepatobiliary Surgery Department of the Fifth Medical Center of the General Hospital of the People’s Liberation Army between October 2016 and May 2023 and aged over 18 years.
2.2. Clinical diagnosis of MCN, obtained through the medical record retrieval system.
3.3. Consistent diagnosis of MCN confirmed by three experienced pathologists through examination of routine pathological slides.

## Methods

The study was approved by the Medical Ethics Committee of the Fifth Medical Center of the General Hospital of the PLA. The research was carried out retrospectively, using anonymous data. As a result, the Medical Ethics Committee of the Fifth Medical Center of the General Hospital of the PLA has approved the exemption of obtaining informed consent. All the experiments were performed according to relevant guidelines and regulations. Clinical data from seven patients were collected using the medical record retrieval system, including general information, clinical manifestations, laboratory tests, imaging examinations, surgical methods, and postoperative routine pathological results. Statistical analysis was conducted to understand the clinical characteristics and summarize the diagnostic and treatment experience. Table 1 presents their key characteristics.

**Table 1:** Primary data from seven cases of MCN.

No	Age (yrs)	Gender	Symptom	CA 19-9 serum level (0–37)^a^, U/mL	Lesion diameter (cm)	Lesion location	Operative procedure	Pathology	Follow-up status
1	55	Female	Abdominal pain	> 1000	2.8	Left lobe	Left hepatectomy	Invasive carcinoma	The patient experienced tumor recurrence 9 month post-surgery and tragically passed away 2 weeks laters
2	56	Female	Right upper quadrant pain	201	14.6	Left lobe	Cyst fenestration procedure	Mucinous cystic tumor	Alive
3	23	Male	Right upper quadrant pain	94	13.1	Right lobe	Right hemihepatectomy with caudate lobe	Mucinous cystic tumor	Alive
4	36	Female	Abdominal pain	425	16.7	Left lobe	Left hepatectomy	Mucinous cystic tumor	Alive
5	44	Female	Asymptomatic	0.6	19.8	Left lobe	Left hepatectomy	Mucinous cystic tumor	Alive
6	21	Female	Fever, Jaundice	22.6	3.3	Left lobe	Left hepatectomy	Mucinous cystic tumor	Alive
7	46	Female	Abdominal pain	11.27	7.8	Right, left lobe	Extended left hepatectomy	Invasive carcinoma	Alive

^a^ represents that the range of 0–37 in the brackets is the normal reference range for the CA199 test.

### Statistical analysis

Data are presented as mean ± standard deviation. Statistical analysis included t-tests for comparing means between groups and the chi-square test for comparing categorical data. SPSS ver. 13.0 statistical software (SPSS, Chicago, IL, USA) was used for all calculations. Statistical significance was determined at a significance level of *P* < 0.05.

## Results

### General information

The study included 7 cases of MCN confirmed by postoperative pathological examination. Among these, 5 were diagnosed as mucinous cystic tumors and 2 as invasive carcinomas. Except for one patient with concurrent hepatitis C virus infection and invasive carcinoma, the remaining patients had no history of viral hepatitis. Tumor diameters ranged from 3.3 to 20 cm, with an average diameter of 10.2 cm. MCN did not have specific clinical manifestations. During examination, 5 individuals reported symptoms of abdominal distension and pain. One individual had fever and jaundice, while another was asymptomatic and incidentally discovered a liver mass during routine medical check-up.

### Laboratory examinations

Among the cases, one patient had positive test result for anti-HCV antibodies, indicating concurrent hepatitis C infection. Furthermore, elevated CA19-9 levels were observed in three cases. However, the liver and kidney function tests and other tumor markers of the remaining patients were within normal ranges.

### Radiological examinations, preoperative diagnoses, and surgical procedures

**Figure 1:**
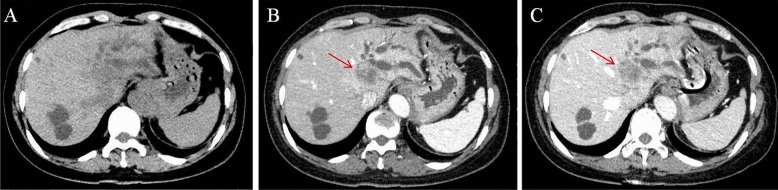
(Patient 1) Abdominal contrast-enhanced CT scan reveals a 2.8 * 2.5 cm slightly low-density circular lesion in the left lobe of the liver. Mild enhancement is seen at the lesion edges during the arterial phase, while mild infiltration of the contrast agent leads to slightly lower-density appearance in the portal venous phase. Adjacent left hepatic lobe shows bile duct dilation. Images (**A**), (**B**), and (**C**) correspond to the non-contrast, arterial, and venous phases, respectively.

**Figure 2:**
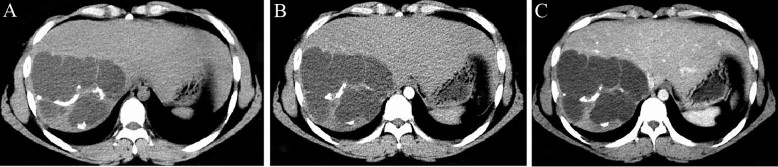
(Patient 3) Abdominal contrast-enhanced CT scan shows a non-enhancing, circular, fluid-density lesion with multiple septations and partial calcifications in the non-contrast phase. The contrast-enhanced scan reveals enhancement confined to the relatively thick septations within the lesion.

**Figure 3:**
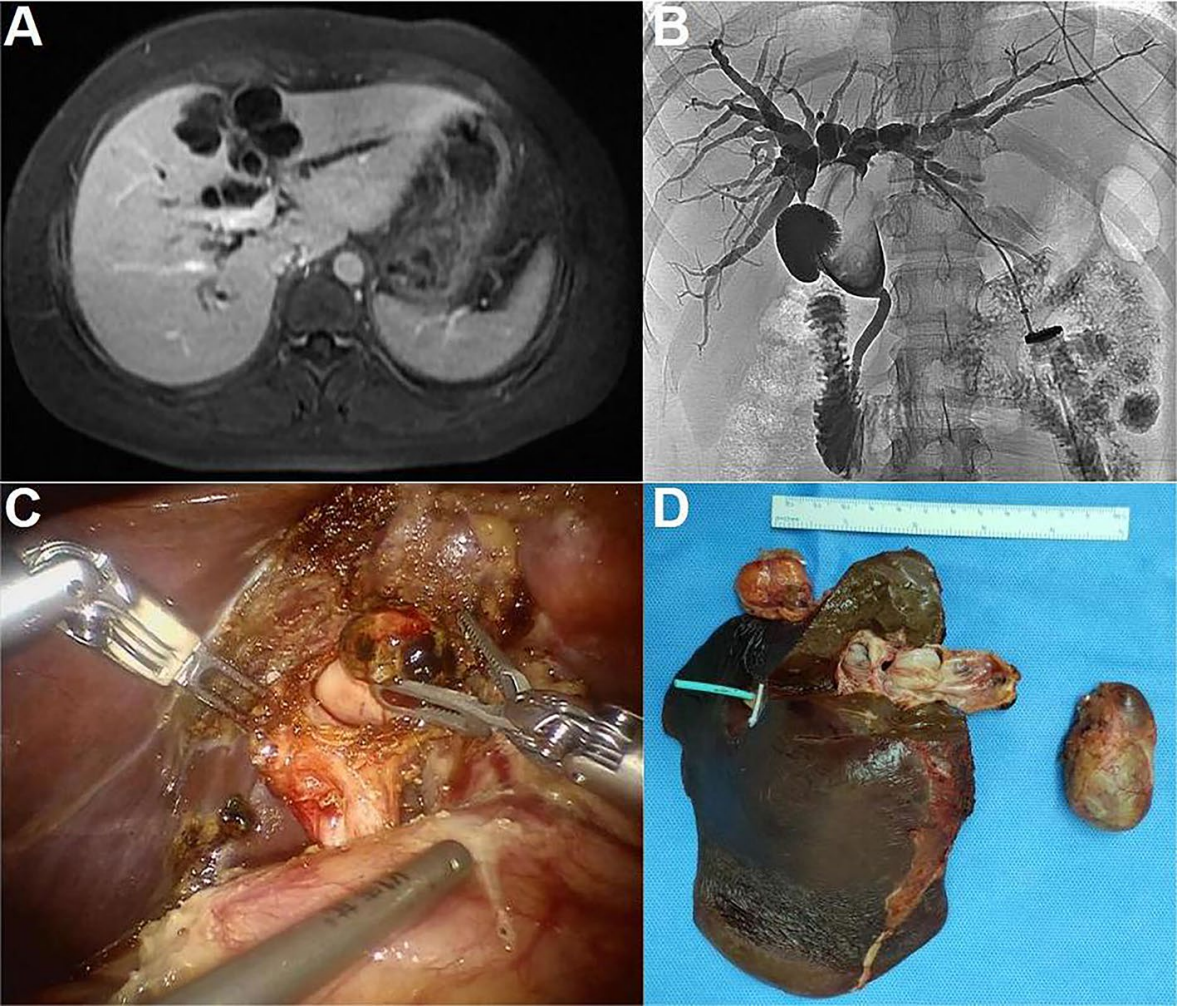
(Patient 6) Image (**A**) shows multiple circular liver lesions with long T1 and long T2 signals, some with septations. The largest lesion, located in the left lobe of the liver, communicates with the intrahepatic bile duct and measures approximately 3.3 × 2.6 cm. Image (**B**) shows a cystic filling defect in the common bile duct. Image (**C**) displays an intraoperative view of a cystic outgrowth extending from the left hepatic duct into the common bile duct. Image (**D**) presents the resected specimen of the gallbladder and left lobe of the liver.

**Figure 4:**
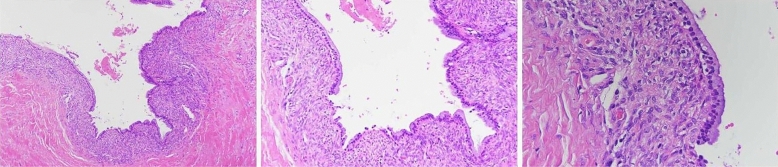
(Patient 6) The pathological image shows a lining of tall columnar cells, with areas of cuboidal and squamous cells, and cellular-rich ovarian-like stroma.

Patient 1: Diagnosed with hepatobiliary ductal carcinoma (Fig. 1). Underwent left hemihepatectomy. Pathology revealed invasive carcinoma associated with mucinous cystic tumor. Patient 2: Underwent laparoscopic fenestration and drainage for liver cyst. Pathology revealed mucinous cystic tumor. Subsequently, underwent left hepatectomy, confirming the diagnosis. Patient 3: Diagnosed with mucinous cystic tumor (Fig. 2). Underwent right hepatectomy with partial resection of the caudate lobe. Pathology confirmed the diagnosis. Patient 4: Preoperatively diagnosed with cystic mass in the left lobe. Underwent left hepatectomy. Pathology confirmed mucinous cystic tumor. Patient 5: Preoperatively diagnosed with large cystic mass in the left lobe. Underwent left hepatectomy. Pathology confirmed mucinous cystic tumor. Patient 6: Experienced recurrence of hepatic cyst after laparoscopic fenestration and drainage. Initially misdiagnosed as a bile duct cyst (Fig. 3A). Percutaneous transhepatic cholangiodrainage (PTCD) revealed cystic filling defect in the common bile duct (Fig. 3B). Robotic left hemihepatectomy exposed cystic outgrowth extending into the bile duct (Fig. 3C,D). Pathology confirmed mucinous cystic tumor (Fig. 4). Patient 7: Preoperatively diagnosed with cystic mass at the junction of left and right lobes, suspected malignancy. Underwent extended left hepatectomy. Pathology confirmed invasive carcinoma associated with mucinous cystic tumor.

### Tumor location and pathological examination

In the study, five tumors were found in the left lobe of the liver, one in the right lobe, and one at the junction of the left and right lobes. Pathologically, five cases were diagnosed as mucinous cystic tumors, while the remaining two were identified as invasive carcinoma.

### Follow-up status

All seven patients in this study underwent follow-up assessments for a period ranging from 3 to 60 months. Regrettably, one patient experienced tumor recurrence 9 months after surgery and passed away 2 weeks later. However, the remaining six patients are still alive, with survival times ranging from 3 to 60 months.

## Discussion

Mucinous cystic neoplasms mainly occur in middle-aged or elderly females over 40, with less frequency in younger populations. Invasive carcinoma, on the other hand, can affect individuals of all age groups, being more common in children and young adults and less prevalent in the elderly^15–17^. This study included 1 male and 6 females, aged 21–56 years (mean age: 42).

Mucinous cystic neoplasms are typically asymptomatic initially but can cause symptoms such as abdominal distension, pain, indigestion, and nausea as they grow or complications arise. These symptoms are nonspecific, but the presence of a mass or cyst during examination should be a cause for concern^18–20^. In contrast, invasive carcinoma often presents early symptoms including abdominal pain, fatigue, loss of appetite, weight loss, jaundice, nausea, and vomiting. These symptoms are usually more severe and may be accompanied by abnormal liver function or liver cirrhosis^2–5^. In this study, one patient with asymptomatic mucinous cystic neoplasms was incidentally detected during a physical examination. Among the patients, three with mucinous cystic neoplasms and two with invasive carcinoma experienced abdominal pain. Notably, a patient with mucinous cystic neoplasms who had undergone liver cyst fenestration surgery developed fever and jaundice after recurrence.

Mucinous cystic tumors often have elevated CA19-9 and CEA levels, while invasive carcinoma can cause increased levels of multiple tumor markers like CA19-9, CEA, and more^9,21–24^. Fuks et al. found that CA19-9 levels could potentially differentiate between mucinous cystic tumors and invasive carcinoma before surgery. Among 27 patients with bile duct invasive carcinoma, 19 showed a significant increase in CA19-9, suggesting its potential utility in distinguishing between mucinous cystic tumors, invasive carcinoma, and other liver conditions^9,25^. In this study, CA19-9 levels were elevated in two patients with Mucinous cystic tumors, with more significant increase seen in one patient with invasive carcinoma.

Ultrasound, CT, and MRI are the main imaging methods used for preoperative diagnosis of MCN. These modalities offer valuable information on tumor location, size, and characteristics^26,27^. We obtained the diagnosis of cases shown in Figs. 1 and 2 through abdominal contrast-enhanced CT scans. This scanning method provides information about the morphology, size, density, and enhancement features of the lesions. CT imaging of mucinous cystic tumors typically shows low-density cystic masses in the liver with single or multiple cystic changes. These cysts have thin walls, and their CT enhancement value is generally less than 30 HU. In cases of multiple cysts, partitions between them enhance on contrast-enhanced CT scans, while the contents within the cysts do not enhance^28^. On MRI, the lesions appear as low signal intensity on T1-weighted images and high signal intensity due to the presence of cystic fluid on T2-weighted images. The signal intensity can vary depending on factors such as the nature of the cystic fluid, protein concentration, and intracystic hemorrhage. Preoperative MRCP can provide valuable information about the location of bile duct obstruction, aiding in the determination of the appropriate surgical approach^8,9,27^. Mucinous cystic tumors shows similar CT and MR imaging features, including irregular cystic cavities, thickened cyst walls, and nodular enhancement of the cyst walls. The presence of nodules within the cyst walls and cyst wall calcifications are important diagnostic indicators for distinguishing bile duct invasive carcinoma^23^. To diagnose challenging MCN accurately, comprehensive PET-CT examination can be considered. PET-CT combines metabolic and anatomical imaging, enabling the assessment of both the lesion's metabolic activity and potential malignancy^29^.

In this study, two cases initially misdiagnosed as simple cysts were later correctly identified as mucinous cystic tumors. Therefore, early surgical resection is recommended for the following reasons. Firstly, it helps prevent malignant transformation as mucinous cystic tumors have potential risk of malignancy. Early surgical resection reduces this risk and improves the chances of cure. Secondly, even though they may appear as simple cysts, mucinous cystic tumors can still recur or progress after surgery. Early surgical resection ensures complete removal of the tumor, reducing the risk of recurrence and progression. Lastly, surgical resection provides definitive diagnosis and detailed pathological evaluation. For misdiagnosed cases of mucinous cystic tumors, the surgical procedure confirms the diagnosis, rules out other possible pathological types, and enables more accurate prognosis assessment. In summary, early surgical resection effectively manages preoperatively misdiagnosed mucinous cystic tumors, lowering the risks of malignant transformation, recurrence, and progression while providing a definitive diagnosis and detailed pathological evaluation, ultimately improving the success rate of treatment.

Clinicians should consider the possibility of MCN when diagnosing liver cysts. For patients with progressive cyst enlargement, abdominal pain, and distension, a comprehensive evaluation should include imaging and serum markers. While histopathology and cytology can assist in tumor diagnosis, caution is necessary with tumor puncture to avoid potential risks. The authors emphasize the importance of careful consideration before performing tumor puncture prior to surgery.

Surgical resection is the main treatment for MCN and can lead to positive prognosis for patients with mucinous cystic tumor^30^. Anatomical liver resection or hemihepatectomy can be performed for localized lesions with adequate remaining liver volume. Complete tumor excision is possible for centrally located lesions or those near major blood vessels. In cases where the lesion communicates with the bile duct near the hepatic hilum, regular intraoperative cholangioscopy and consideration of bile duct resection and reconstruction are recommended. To ensure sufficient clearance, expanding the scope of liver resection by more than 2 cm is advised for suspected malignancy. Intraoperative pathological examination of regional lymph nodes and the lesion should be considered to determine the need for lymph node dissection. Robotic surgical resection yielded favorable outcomes in one of seven patients, improving postoperative recovery, reducing hospital stay, and increasing patient acceptance compared to traditional surgery. However, invasive carcinoma, which is malignant condition, usually has worse prognosis due to higher risk of recurrence and metastasis. Liver transplantation may be considered as a treatment option for unresectable tumors or patients with underlying liver disease. However, this approach is typically reserved for highly selected cases and requires a comprehensive patient evaluation and the availability of suitable donor organs^31^.

This study holds significant potential in the diagnosis and treatment of MCN. Through retrospective analysis and an extensive literature review, it thoroughly explores the clinical characteristics, diagnostic methods, and treatment strategies for MCN. The researchers conducted a comprehensive study involving seven patients diagnosed with MCN, shedding light on the feasibility and effectiveness of surgical resection as a prominent treatment modality for this condition. Their findings offer valuable insights into the potential of surgical intervention in managing MCN and contribute to the existing knowledge in this field. However, this study also revealed certain knowledge gaps. Due to the absence of distinct clinical and radiological features, MCN is susceptible to misdiagnosis and inappropriate treatment. Consequently, accurately diagnosing MCN and selecting appropriate treatment methods remain challenging. The researchers also emphasized the importance of identifying prognostic biomarkers to guide treatment decisions. To address these knowledge gaps, future research can focus on the following areas. Firstly, further investigation of the clinical manifestations and radiological features of MCN is needed to enhance diagnostic accuracy. Secondly, conducting larger-scale clinical studies to validate the feasibility and long-term efficacy of surgical resection in treating MCN is crucial. Additionally, molecular biology and genetics research can be pursued to identify biomarkers associated with MCN development and prognosis.

Over the next 5 years, we anticipate continuous advancement in this field. With a deeper understanding of MCN and technological advancements, we can expect significant progress in the early diagnosis and treatment of MCN. This progress may lead to the emergence of more accurate diagnostic methods and personalized treatment strategies, ultimately improving survival rates and enhancing the quality of life for patients. Furthermore, as molecular biology and genetics continue to evolve, we can anticipate the utilization of an expanded range of biomarkers for prognostic assessment and treatment selection. Overall, we hold an optimistic outlook for the future development of this field and eagerly anticipate further research outcomes and clinical applications.

The study has several limitations, including a small sample size, single-center design, retrospective approach, absence of a control group, limited follow-up duration, and a lack of molecular biology analysis. Future research endeavors should aim to address these limitations by incorporating larger sample sizes, adopting a multicenter design, and integrating research methodologies from the field of molecular biology. These enhancements will enable a more comprehensive exploration of the diagnosis and treatment strategies for MCN.

## Conclusions

In conclusion, MCN of the liver present a diagnostic challenge due to the lack of distinct clinical and radiological features, potentially leading to misdiagnosis and inappropriate treatment. Therefore, consideration of MCN should be given when evaluating patients with suspected liver cystic diseases. Surgical resection has demonstrated favorable therapeutic outcomes for patients with MCN. However, it is important to note the possibility of tumor recurrence and metastasis, emphasizing the need for close postoperative follow-up and surveillance. Further research is necessary to enhance our understanding of the clinical characteristics, diagnostic approaches, and treatment strategies for liver MCN. This will contribute to improved efficacy in diagnosis and treatment of this rare liver tumor.

## Data Availability

If anyone requires access to the raw data, please feel free to contact us. The data may be used for scientific research, subject to appropriate permissions and regulations. The data presented in this study can be obtained upon reasonable request from the corresponding author.
